# Factoring Prior Treatment into Tuberculosis Infection Prevalence Estimates, United States, 2011–2012

**DOI:** 10.3201/eid2510.190439

**Published:** 2019-10

**Authors:** Laura A. Vonnahme, Maryam B. Haddad, Thomas R. Navin

**Affiliations:** Centers for Disease Control and Prevention, Atlanta, Georgia, USA

**Keywords:** Prevalence, surveys and questionnaires, tuberculosis and other mycobacteria, United States, tuberculosis, TB, bacteria, prior treatment

## Abstract

To refine estimates of how many persons in the United States are candidates for treatment of latent tuberculosis, we removed from analysis persons who self-reported prior treatment on the National Health and Nutrition Examination Survey 2011–2012. We estimate that 12.6 million persons could benefit from treatment to prevent active tuberculosis.

In the United States, although tuberculosis (TB) incidence is at historic lows, the average annual rate of decline has slowed to 2% (*1*). To achieve TB elimination by 2100, sustained annual declines of twice that magnitude are needed (*2*). Most new cases of TB in the United States result from progression of *Mycobacterium tuberculosis* infections acquired years earlier (*3*). TB elimination requires scaling up treatment of latent TB infection (LTBI) to prevent progression to active TB. The National Health and Nutrition Examination Survey (NHANES) 2011–2012 determined that ≈5% of the US population had LTBI on the basis of positivity of a tuberculin skin test (TST) or an interferon-γ release assay (IGRA) (*4*). However, these TB test results may remain positive even after a patient has received effective treatment for active TB or LTBI. Specifically, the TST is widely believed to remain positive for life; whether the IGRA blood test remains positive is still undetermined (*5*–*7*). To better estimate the number of persons in the United States who are candidates for LTBI treatment (*8*), we refined the NHANES-based estimate of the national LTBI prevalence by excluding from analysis persons who reported having received prior treatment for active TB or LTBI.

## The Study

The only nationally representative survey that reflects both TST and IGRA results is the NHANES 2011–2012. NHANES cross-sectional surveys are implemented in consecutive 2-year cycles and are designed to assess the health and nutritional status of the civilian, noninstitutionalized US population (*9*). To obtain this nationally representative sample, NHANES uses complex, stratified, multistage probability cluster sampling (*10*). The survey consists of questionnaires administered in the home, followed by a medical examination conducted in a mobile examination center. The NHANES 2011–2012 questionnaire included questions about participants’ history of TB testing and diagnosis and any prior treatment for active TB or LTBI (*9*). The medical examinations included a TST and an IGRA for TB infection for participants >6 years of age (*4*).

We defined a positive test result for TB infection as a positive result for an IGRA administered by NHANES according to manufacturer’s standards (*11*) or a TST reaction of >10 mm. Self-reported prior treatment was defined as a participant’s answer of yes to NHANES questions about having ever been prescribed medicine for TB disease or to keep from getting sick with TB (i.e., LTBI treatment). We ascertained prevalence estimates of self-reported prior treatment among persons >6 years of age who had a positive result for TB infection for a test conducted by NHANES. We assessed treatment history among subgroups with IGRA positivity, TST positivity, TST or IGRA positivity, or dual TST and IGRA positivity. For comparison, we also assessed treatment history among persons with dual TST and IGRA negativity. We stratified subgroups by birthplace. We calculated population prevalence estimates and corresponding 95% CIs by using SAS software (https://www.sas.com), which accounted for the complex survey design, the 2-year examination weights, and population denominators from the 2011 American Community Survey data.

All NHANES participants or their proxies provided informed consent, and the Research Ethics Review Board of the National Center for Health Statistics reviewed all procedures and protocols. All data used for this analysis are publicly available (https://www.cdc.gov/nchs/nhanes).

Having already been treated for TB disease or for LTBI was self-reported by 12.2% (95% CI 8.5%–15.8%) participants, 1.8 (95% CI 1.1–2.3) million of the 14.1 (95% CI 11.9–16.4) million persons in the civilian, noninstitutionalized US population with a positive IGRA blood test result for TB infection. This finding suggests that as of 2011–2012, 12.6 (95% CI 10.5–14.7) million persons could still benefit from LTBI treatment after further evaluation (i.e., after obtaining radiologic evidence to help exclude a diagnosis of active TB disease). 

Stratification by birthplace did not reveal substantial differences for prevalence of prior treatment for TB disease or LTBI; 11.3% (95% CI 5.5%–17.1%) of persons born in the 50 US states or the District of Columbia (US-born) and 13.0% (95% CI 9.5%–16.5%) of non–US-born persons with a positive IGRA result reported having received prior treatment (Figure). Excluding those persons, an estimated 6.5 (95% CI 4.4–8.5) million US-born persons and 5.5 (95% CI 4.6–6.3) million non–US-born persons with a positive IGRA result reported no prior treatment.

**Figure:**
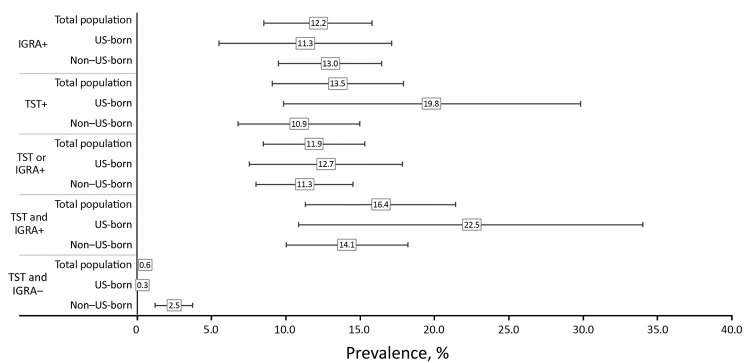
Estimated prevalence of previous tuberculosis treatment among persons tested for tuberculosis infection, United States, 2011–2012. Boxes represent prevalence estimates and corresponding horizontal lines represent 95% CIs. IGRA, interferon-γ release assay; TST, tuberculin skin test; +, positive; −, negative.

When also considering TST results in the definition of a positive test result for TB infection, the prevalence of self-reported prior TB treatment among persons with TB infection ranged from 11.9% to 16.4%, with overlapping CIs (Figure). Among subgroups, the prevalence range for prior treatment was wider overall among US-born persons (11.3%–22.5%) than among non–US-born persons (10.9%–14.1%); the highest rate of self-reported prior TB treatment was among US-born persons with dual positive results for the TST and the IGRA: 22.5% (95% CI 10.9%–34.0%).

Among persons with dual negative results for the TST and IGRA, the prevalence of prior TB treatment was 0.6% (95% CI 0.3%–0.9%). Prevalence was higher among non–US-born (2.5% [95% CI 1.2%–3.7%]) than among US-born (0.3% [95% CI 0.1%–0.6%]) persons.

## Conclusions

An estimated 12.6 (95% CI 10.5–14.7) million persons living in the United States with evidence of TB infection by IGRA result reported no prior TB treatment. This number excludes ≈12% of persons in previously reported estimates of the number of persons with LTBI in the United States (*4*). In estimating the potential effect of interventions to expand screening and treatment for LTBI, our estimate of 12.6 million untreated TB-infected persons is a more meaningful measure for determining potential individual and societal benefits of LTBI treatment.

A limitation of this analysis is that previous medication history was self-reported only. Recall bias might have resulted in the misreporting of previous LTBI or TB treatment. In addition, we cannot assume that all persons who reported prior treatment completed the regimen, although partial treatment of TB has been shown to be effective (*12*). However, because the questionnaire was administered several days before the medical examination, knowledge of the outcome of the NHANES test for *M. tuberculosis* infection would not have influenced this response. A treatment adherence question was included in the survey; however, a positive response to the question was not used to define prior treatment because it pertained only to completing treatment for LTBI, and >90% reported treatment completion. In addition, a single 2-year NHANES cycle is not designed to provide stable prevalence estimates for detailed subpopulations (e.g., non–US-born persons with a certain test result); consecutive NHANES cycles of at least 4 or 6 years would provide more precise estimates (*8*,*9*).

The higher prevalence of self-reported prior treatment among persons with a positive test result for *M. tuberculosis* infection helps validate the 2 implicit assumptions that anyone who had received prior treatment would have had at that time a positive test result (or a diagnosis of active TB disease) and that the result would have remained positive at the time of the NHANES examination. Conversely, the <1% prevalence of prior treatment history among those with negative TST and IGRA results suggests that some test results became negative after treatment or that some uninfected participants inaccurately recalled having taken medication for active TB disease or LTBI.

Regardless of which diagnostic test for TB infection was considered, consistently ≈12% of those with positive results during the NHANES 2011–2012 self-reported prior TB treatment. In conclusion, we estimate that the other 88% (i.e., ≈6.5 million US-born persons and ≈5.5 million non–US-born persons living in the United States) could benefit from interventions to expand screening and treatment for LTBI.
